# Cost-effectiveness analysis of rapid diagnostic test, microscopy and syndromic approach in the diagnosis of malaria in Nigeria: implications for scaling-up deployment of ACT

**DOI:** 10.1186/1475-2875-8-265

**Published:** 2009-11-23

**Authors:** Benjamin SC Uzochukwu, Eric N Obikeze, Obinna E Onwujekwe, Chima A Onoka, Ulla K Griffiths

**Affiliations:** 1 Department of Community Medicine, College of Medicine, University of Nigeria, Enugu-campus, Nigeria; 2 Health Policy Research Group, College of Medicine, University of Nigeria, Enugu-campus, Nigeria; 3 Department of Health Administration and Management, College of Medicine, University of Nigeria, Enugu-campus, Nigeria; 4 Health Policy Unit, London School of Hygiene and Tropical Medicine, London, UK

## Abstract

**Background:**

The diagnosis and treatment of malaria is often based on syndromic presentation (presumptive treatment) and microscopic examination of blood films. Treatment based on syndromic approach has been found to be costly, and contributes to the development of drug resistance, while microscopic diagnosis of malaria is time-consuming and labour-intensive. Also, there is lack of trained microscopists and reliable equipment especially in rural areas of Nigeria. However, although rapid diagnostic tests (RDTs) have improved the ease of appropriate diagnosis of malaria diagnosis, the cost-effectiveness of RDTs in case management of malaria has not been evaluated in Nigeria. The study hence compares the cost-effectiveness of RDT versus syndromic diagnosis and microscopy.

**Methods:**

A total of 638 patients with fever, clinically diagnosed as malaria (presumptive malaria) by health workers, were selected for examination with both RDT and microscopy. Patients positive on RDT received artemisinin-based combination therapy (ACT) and febrile patients negative on RDT received an antibiotic treatment. Using a decision tree model for a hypothetical cohort of 100,000 patients, the diagnostic alternatives considered were presumptive treatment (base strategy), RDT and microscopy. Costs were based on a consumer and provider perspective while the outcome measure was deaths averted. Information on costs and malaria epidemiology were locally generated, and along with available data on effectiveness of diagnostic tests, adherence level to drugs for treatment, and drug efficacy levels, cost-effectiveness estimates were computed using TreeAge programme. Results were reported based on costs and effects per strategy, and incremental cost-effectiveness ratios.

**Results:**

The cost-effectiveness analysis at 43.1% prevalence level showed an incremental cost effectiveness ratio (ICER) of 221 per deaths averted between RDT and presumptive treatment, while microscopy is dominated at that level. There was also a lesser cost of RDT ($0.34 million) compared to presumptive treatment ($0.37 million) and microscopy ($0.39 million), with effectiveness values of 99,862, 99,735 and 99,851 for RDT, presumptive treatment and microscopy, respectively. Cost-effectiveness was affected by malaria prevalence level, ACT adherence level, cost of ACT, proportion of non-malaria febrile illness cases that were bacterial, and microscopy and RDT sensitivity.

**Conclusion:**

RDT is cost-effective when compared to other diagnostic strategies for malaria treatment at malaria prevalence of 43.1% and, therefore, a very good strategy for diagnosis of malaria in Nigeria. There is opportunity for cost savings if rapid diagnostic tests are introduced in health facilities in Nigeria for case management of malaria.

## Background

Malaria is the number one cause of mortality and morbidity in Nigeria and accounts for 25 and 30% of infant and childhood deaths, respectively and 11% maternal mortality [1]. Most victims of malaria still die because the disease is not diagnosed in time by health workers [2]. The diagnosis of malaria has traditionally relied on the clinical presentation of malaria symptoms [3,4] and microscopical examination of Giemsa-stained blood films. Diagnosis based on symptoms alone is unreliable because the symptoms of malaria are non-specific, overlapping with other febrile diseases [5]. Studies in Africa have shown that more than 50% of patients clinically diagnosed with malaria have illnesses attributable to some other causes [6-8]. This results in over-diagnosis of malaria [9], over-prescription of anti-malarial drugs, under-diagnosis and inappropriate treatment of non-malarial febrile illnesses (NMFI) [10-14]. It is also costly and associated with side-effects [6] and ultimately contributes to the development and spread of drug resistance [7,15,16].

Although microscopy is considered to be the gold standard for malaria diagnosis [16,17], in many malaria-endemic areas like Nigeria, there is lack of trained microscopists and reliable equipment [18].

As anti-malarial drug costs increase, diagnostic methods are becoming a crucial component of malaria control and prevention. Treating all fevers with anti-malarial medications will no longer hold with the introduction of a higher-priced artemisinin-based combination therapy (ACT), which was introduced in Nigeria in 2005 as the first-line anti-malarial drug, as a result of extensive resistance to chloroquine and sulphadoxine-pyrimethamine (SP) [19]. It has been noted that the cost of ACT is up to ten times more than chloroquine [20]. Although the prices of ACT have reduced recently, the recommended ACT (artemether-lumefantrine) in Nigeria is still sold at $6 to $8. Thus with the high cost of treatment for malaria, there is an increased need to ensure that malaria is correctly diagnosed prior to treatment [21].

Developments in rapid diagnostic tests (RDTs) based on the demonstration of parasite antigens have opened new possibilities for improved remote malaria diagnosis that is independent of microscopic diagnosis [18,22-25]. Several commercially available tests are sensitive, specific, and stable under operational conditions [14,26,27]. WHO recommended that parasite-based diagnosis should be used in all cases of suspected malaria with the possible exception of children in high-prevalence areas and in certain other situations [28]. RDTs can be performed close to home in settings with no sophisticated infrastructure, and they do not require much skill although some level of training is needed in order for RDTs to be used properly.

In spite of these advances, there is paucity of cost-effectiveness data of RDTs in highly malaria endemic countries like Nigeria. Although studies have investigated the cost-effectiveness of RDT in Africa [14,28-30], little is known on its cost-effectiveness in Nigeria. This paper reports an economic evaluation carried out to determine whether the use of RDT for diagnosis of malaria is cost-effective, when compared with clinical diagnosis and microscopy-based diagnosis of malaria. The cost-effectiveness analysis is based on the model by Shillcutt *et al *[28], with data on cost and malaria epidemiology obtained from south-east Nigeria. The information generated by this study will help design policy measures to strengthen the diagnosis and treatment components of the national malaria control strategy especially in the light of the introduction of ACT in Nigeria.

## Methods

### Study area

The study was undertaken in urban and rural districts of Enugu East Local Government Area in Enugu State, south-east Nigeria, with a 2006 population of 279,089 [31]. It has 12 public health centres and 30 private clinics and hospitals. The health centres are stratified into three groups of high, medium and low level of infrastructures based on the number of staff, availability of relevant facilities, such as maternity beds and utilization rates. All the centres have drug dispensing units, but no laboratory facilities. There is all year high transmission rate of malaria in the study area. Patients were prospectively recruited over a 24-month period (between 2005 to 2007) in order to account for seasonal variations in malaria occurrence.

### Overall study design and model used for CEA

**Figure 1:**
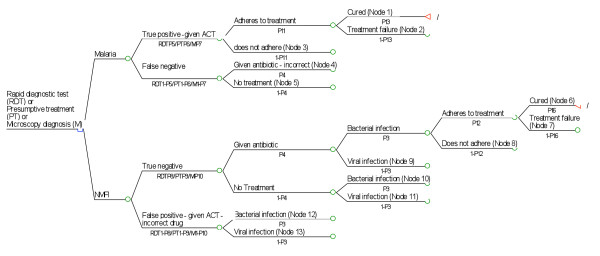
**Root decision tree**.

A decision tree model (Figure 1) was used for cost-effectiveness analysis using a hypothetical cohort of 100,000 patients. The patients entered the model at the point at which a decision is made between use of symptoms only, a rapid diagnostic test or microscopy for diagnosis of malaria in a patient presenting to a health worker with symptoms suggestive of malaria. Real-life data on cost and malaria epidemiology were locally obtained from out-patients attending health clinics. The estimation of effectiveness was done based on the model by Shillcutt *et al *[28] and along with the cost and epidemiology data, available evidence on effectiveness was used to populate the model. Figure 1 illustrates the decision tree that starts with patients who come to the health facility with malaria symptoms. This proceeds through diagnosis and treatment to disease outcomes depending on the sensitivity and specificity of each diagnostic strategy. Presumptive diagnosis and treatment of patients served as the base intervention.

### Sampling and sample size

Four out of the six districts with at least one highly or medium developed health centre each (two rural and two urban) were purposively chosen. In each of the chosen district, one health centre was chosen as a base clinic for the study. All patients presenting at the centres with history of fever or non-specific symptoms or signs suggestive of malaria (e.g. headache only, chills, rigors) and diagnosed clinically as having malaria by the health facility personnel were recruited for the study. However, those who have taken anti-malarial drugs, and those refusing to participate were excluded from the study.

The required number of blood smear positive patients was calculated according to the following parameters: expected sensitivity of microscopic test = 90%, precision = 5% and alpha error = 0.05. This gave a sample size of 136. This was doubled taking into account a stratified analysis by age group category (0-4 years and 5 years and above). Similar parameters (with expected specificity of 90%) were used to calculate the required number of blood smear negative patients. The minimum final sample size was, therefore, fixed at 300 positive and 300 negative subjects, giving a total of 600 individuals.

### Data collection

Patients presenting to the facility were seen by health workers at the facility and those who had symptoms suggestive of malaria had their axillary temperature taken and recorded after which the health worker took a decision as to whether the patient had malaria or not. Those considered to have malaria were recruited for the study. Following an informed consent, information was then obtained from such patients using pre-tested interviewer-administered questionnaire on patient's socio-economic and demographic background, the presenting symptoms, history of treatment, malaria specific knowledge and household practices to diagnose and treat malaria. Patients clinically diagnosed as having malaria had capillary blood samples taken by a single finger puncture by the health workers for both microscopy and RDTs.

Two thick and one thin blood films (as a single slide cannot exclude infection) was prepared from each sample. The microscopical examination of the standard Giemsa-stained blood films was prepared in the field and read by a microscopist. The smears were examined with 100× magnification under oil-immersion objectives.

At the same time, RDTs were conducted using ICT Malaria Combo Cassette Test (ML02)(ICT Diagnostics, Cape Town, South Africa) and read according to the manufacturers' instructions by the facility health workers who were trained over a three-day period by the study coordinators. The sensitivity and specificity of the test in another setting is put at 96% and 95%, respectively [26].

Following diagnosis, patients with a positive result received an artemisinin-based combination therapy (ACT)(dihydroxy-artemisinin/piperaquine). Parasitaemia demonstrated by microscopic tests was used as the "gold standard" to compare symptom-based, microscopy-based and RDT-based diagnosis. All patients who were RDT and microscopy negative were given amoxicillin and referred to a secondary facility for further investigation and management.

Household visits to the patients took place twice weekly for three weeks for a post-diagnosis survey. On the first week, the blister pack at the patient's home was examined for any remaining tablets to assess adherence to ACT. Verbal confirmation of completion of all doses with the presence of an empty blister pack and correct description of how the dose was taken was regarded as complete adherence. On day 21, the clinical outcome of the illness was noted. This was assessed as the presence or otherwise of occurrence of fever and other initial symptoms at any time within the 21-day period following completion of treatment. The post-diagnosis survey included further questions regarding cost of any initial or follow-up treatment, and household assets. Expenditure on drugs and other items was recorded with the aid of pictorial diagrams.

### Data analysis

The three alternatives pathways considered in this analysis for diagnosis and treatment or malaria are symptomatic treatment, treatment following diagnosis with RDT, and treatment following microscopic diagnosis.

### Cost data

Cost data used was based on the consumer and provider perspectives (Table 1). Consumer costs included cost of registration, drug, laboratory, admission and transportation to health facility. Provider costs were those of capital and recurrent items for malaria diagnosis and treatment. These included cost of staff time, training and supervision, unit cost of RDT test kit, cost of consumables, such as lancet, cotton wool, methylated spirit, reagents for microscopy, slides, hand gloves, ACT, amoxicillin, and oral and intravenous quinine. Out-patient costs per visit, in-patient costs per day, costs of transportation, insurance and wastages were also included. Costs of using health centres and hospitals were also differentiated.

**Table 1:** Model Parameter Values

**No**.	Disease Parameter	Estimate	Source
P1	Malaria prevalence	43.1%	Uzochukwu 2008 [42]

P2	Febrile outpatients aged ≥ 5 years		

P3	Proportion of NMFI cases that were bacterial	10%	Shillcutt et al. 2008

P4	Probability that a NMFI received an antibiotic	100%	Uzochukwu 2008

P5	RDT sensitivity	90%	Uzochukwu 2008, Beadle et. al, 1994, Craig et al., 2002, Bell et. al. 2005

P6	Presumptive treatment sensitivity	100%	Uzochukwu. 2008

P7	Microscopy sensitivity	82%	Uzochukwu. 2008, Shillcutt et. al. 2008, Reyburn et al., 2004

P8	RDT specificity	91%	Uzochukwu. 2008, Mueller et al., 2007 Bell et al. 2005, WHO, 2000

P9	Presumptive treatment specificity	0%	Uzochukwu. 2008

P10	Microscopy specificity	87%	Uzochukwu. 2008

P11	Probability of adherence -- ACT	80%	Uzochukwu. 2008, Depoortere et al., 2004, Fogg et al., 2004

P12	Probability of adherence amoxicillin	80%	Assumption

P13	ACT efficacy (for malaria)	95%	Uzochukwu. 2008. Shillcutt et. al., 2008, Lefevre et. al., 2001

**No**	**Disease Parameter**	**Estimate**	**Source**

P14	Amoxicillin efficacy (for malaria)	0%	Assumption

P15	ACT efficacy (for bacterial infection)	0%	Assumption

P16	Amoxicillin efficacy (for bacterial infection)	75%	Shillcutt et. al. 2008

P17	ACT efficacy (for viral infection)	0%	Assumption

P18	Amoxicillin efficacy (for viral infection)	0%	Assumption

	**Treatment seeking behaviour**		

P19	Outpatient visit at a health centre	1-P20	Shillcutt et.al., 2008

P20	Outpatient visit took place in a hospital	32%	Shillcutt et.al., 2008

P21	Patient with severe illness went to hospital for inpatient care after treatment failure	48%	Shillcutt et. al., 2008, McCombie, 1996

P22	Patient with uncomplicated illness returned to clinic for outpatient care after treatment failure	48%	Shillcutt et.al., 2008

P23	Malaria not effectively treated led to severe disease (age ≥ 5)	1%	Shillcutt et. al. 2008

P24	Malaria not effectively treated led to severe disease (age <5)	7.50%	Shillcutt et. al. 2008

P25	Bacterial illness not effectively treated led to severe disease (age ≥ 5)	15%	Shillcutt et. al. 2008

P26	Bacterial illness not effectively treated led to severe disease (age<5)	30%	Shillcutt et. al. 2008

P27	Viral illness not effectively treated led to severe disease (age ≥ 5)	0%	Assumption

P28	Viral illness not effectively treated led to severe disease (age<5)	0%	Assumption

P29	Severe malaria led to neurological sequelae (age ≥ 5)	1.50%	Shillcutt et. al. 2008

P30	Severe malaria led to neurological sequelae (age<5)	3.50%	Shillcutt et. al. 2008

P31	Severe bacterial infection led to neurological sequelae (age ≥ 5)	3.80%	Shillcutt et. al. 2008

P32	Severe bacterial infection led to neurological sequelae (age<5)	2%	Shillcutt et. al. 2008

P33	Inpatient with severe malaria attending an inpatient facility died (all ages)	10%	Shillcutt et. al. 2008

P34	Inpatient with severe bacterial illness attending an inpatient facility died (all ages)	15%	Shillcutt et. al. 2008

P35	Patient with severe malaria that did not return for formal care would die (all ages)	25%	Shillcutt et. al. 2008

P36	Patient with severe bacterial illness that did not return for formal care would die (all ages)	P35	Assumption

### Outcome and effectiveness

Health outcome was measured in terms of deaths averted based on the use of the alternative diagnostic strategies. In other to estimate such an outcome for the diagnostic and treatment options, sensitivity of RDT and microscopy, estimates of adherence, efficacy of the drugs used, and risks of disease progression as well as recovery have been used based on the model by Shillcutt *et al *[28]. The various estimates used to determine outcome are shown in Table 2. This led to the determination of the number of deaths that would be averted by employing the different diagnostic methods.

**Table 2:** Cost estimates used in model

	Diagnostics	Estimate	Source
C1	PT		
C2	RDT	$0.76	Uzochukwu. 2008
C3	Microscopy	$1.30	Uzochukwu. 2008
	**Drugs**		Uzochukwu. 2008
C4	ACT adult dose	$2.50	Uzochukwu. 2008
C5	Amoxicillin	$0.74	Shillcutt et al. 2008
C6	Oral quinine (10 mg/kg every 8 hours for 7 days	3.12	Shillcutt et al., 2008
C7	Intravenous quinine (initial dose-20 mg/kg over 4 hours	$0.55	Primary data- University of Nigeria Teaching Hospital
C8	Intravenous quinine (per day after -- 10 mg/kg every 8 hours	$0.82	Primary data- University of Nigeria Teaching Hospital
C9	Drugs for severe bacterial infection	2*C5	Assumption
	**Cost Weight**		
C10	Children cost as % of cost of adult dose	1.25	Shillcutt et al., 2008
C11	Drug/RDT wastage, transport, insurance		Assumption
C12	RDT training, additional staff time & QC as % of cost	$0.076	Primary data- University of Nigeria Teaching Hospital
	**Outpatient**		
C13	Patient costs outpatient	$0.69	Uzochukwu 2008
C14	Outpatient cost that are fixed (26%)	$0.18	Shillcutt et. al. (2008)
C15	Outpatient costs that are drugs (37%)	$0.26	Shillcutt et. al. (2008)
C16	Health centre facilities (per visit)	$0.8	Uzochukwu 2008
C17	Hospital facilities (per visit)	$3.90	Uzochukwu 2008
	**Inpatient**		
C18	Patient cost for attending inpatient facility (including transport but excluding fees and cost of patient and caretaker time)	$3.00	Uzochukwu 2008
C19	Proportion of inpatient costs that were drugs	17%	Uzochukwu 2008
C20	Proportion of inpatient facility costs that were fixed		
C21	Provider cost: cost of inpatient faciliy per day	$14.15	Nelson et al. 1995, Kirigia et al., 1998, Barnum & Kutzin, 1993
C22	Average length of stay in days as an inpatient when died (all illnesses)	2 days	Primary data- University of Nigeria Teaching Hospital
C23	Average length of stay in days as an inpatient when had severe malaria and recovered.	4.5 days	Primary data- University of Nigeria Teaching Hospital
			
C24	Average length of stay as an inpatient when had severe bacterial infection and recovered	7.5 days	Primary data- University of Nigeria Teaching Hospital

### Cost-effectiveness analysis

The model for cost effectiveness analysis that was used was based on that developed by Shillcutt *et al *[28]. TreeAge software was used to run the model for the hypothetical cohort of 100,000. Based on the values of costs and outcomes determined, cost effectiveness estimates were determined for the alternative strategies. In addition, an incremental cost effectiveness ratio (ICER) was determined for RDT and microscopy with presumptive diagnosis as the base strategy. ICER was computed for RDT and microscopy as the ratio of the difference between their costs and outcomes relative to those of presumptive diagnosis. All costs are presented in US dollars.

### Sensitivity analysis

The parameters considered for sensitivity analysis were malaria prevalence level, proportion of non-malaria febrile illness that are bacterial, sensitivity of RDT and microscopy, and adherence levels to ACT, and cost of RDT, ACT and amoxicillin.

The prevalence level of 43.1% considered indicates the proportion of those clinically diagnosed for treatment who had malaria based on the gold standard test (microscopy). Sensitivity of the diagnostic tests was considered as the extent to which the test correctly identified those who had the disease, and specificity as the extent to which it identified those who did not have the disease.

## Results

### Characteristics of study respondents

As shown in Table 3, of the 638 patients recruited for this study, 317 (49.7%) and 321 (50.3%) were from the urban and rural areas, respectively. The majority of the respondents 423 (66.3%) were above 30 years and more than a third of the ill persons, 244 (38.2%), were below five years of age. A majority of the ill persons, 439 (68.8%), are females. Most of the respondents (81.7%) had one form of education or another. Their occupational status shows that petty traders are 139(21.8%), farmers 129(20.2), civil servants 97(15.2), self-employed 101(15.8%) and 91 (14.3%) are unemployed.

**Table 3:** Socio-demographic characteristics of respondents and ill persons

Variables	Number (%)
**Residence**	
Urban	317 (49.7)
Rural	321 (50.3)

**Age of Respondents (years)**	
Below 30	215 (33.7)
30 and above	423 (66.3)

**Age of sick person (years)**	
Under 5	244 (38.2)
5 -- 15	233 (36.6)
Above 15	161 (25.2)

**Sex of Respondents**	
Female	567 (88.9)
Male	72 (11.1)

**Sex of ill person**	
Female	439 (68.8)
Male	199 (31.2)

**Marital status**	
Ever married	538 (84.3)
Single	100 (15.7)

**Educational status**	
No education	117 (18.3)
Primary	207 (32.5)
Secondary	285 (44.7)
University	29 (4.5)

**Occupational status**	
Farmer	129 (20.2)
Unemployed	91 (14.3)
Civil servant	97 (15.2)
Petty trading	139 (21.8)
Self employed	101 (15.8)
Big business man/woman	19 (3.0)
Private employment	4 (0.6)
Others	30 (4.7)

### Microscopy and RDT tests results

Out of 638 patients recruited, 275 (43.1%) had microscopic examination of blood smear positive for malaria parasites, 238 (37.3%) were positive for RDT, 224 (35.1%) were positive for both microscopy and RDT, 13 (2.04%) were positive for RDT but negative for microscopy, 49 (7.7%) were positive for microscopy but negative for RDT and 352 (55,2%) were negative for both microscopy and RDT. The mean total patient cost (Drugs, consultation, registration) was $2.52 (SD = $3.63), RDT cost was $0.76 and microscopy $1.30.

### Base case result at malaria prevalence level of 43.1%

The cost-effectiveness analysis at this prevalence level showed an incremental cost effectiveness ratio (ICER) of $221 per death averted between RDT and syndromic treatment while microscopy is dominated at that level (Table 4). For the patient cohort of 100,000, there is also a lesser cost of RDT ($0.34 million) compared to presumptive treatment ($0.37 million) and microscopy ($0.39 million) with effectiveness values of 99,862, 99,735 and 99,851 deaths averted for RDT, presumptive treatment and microscopy respectively.

**Table 4:** Base Case Result at Malaria Prevalence level of 40%

Baseline Cohort of 100,000 Malaria Cases from the TreeAge Result						
**Strategy**	**Cost($)**	**Incremental cost ($)**	**Effect**	**Incremental effect**	**Death Averted**	**Incremental cost per death averted ($)**

Presumptive treatment	365,426		99,735			
RDT	337,466	-27,960	99,862	127	4	-221
Microscopy	394,247	28,821	99,851	116	4	257

### Sensitivity analysis

#### Different prevalence level from 20% to 60%

ICER was sensitive to deterministic values of malaria prevalence. Table 5 shows that for all levels of prevalence that are less than 40%, RDT was more cost effective than microscopy and syndromic approach. Further analysis also showed that at prevalence rate of 20% and 30%, RDT still has most attractive values both in cost and effect while microscopy is dominated at all levels.

**Table 5:** Sensitivity Report by Different Prevalence Level from 20% to 60%

P1	Strategy	Cost	Incr Cost	Eff	Incr Eff	C/E	Incr C/E (ICER)
0.2	RDT	$329,823		99,836		$3	
	M	$388,888		99,826		$4	(Ext Dom)
	PT	$390,737	$60,914	99,662	174	$4	$350
							
0.3	RDT	$333,644		99,849		$3	
	PT	$378,081	$44,437	99,699	150	$4	$296
	M	$391,568		99,839		$4	(Dominated)
							
0.4	RDT	$337,466		99,862		$3	
	PT	$365,426	$27,959	99,735	127	$4	$221
	M	$394,247		99,851		$4	(Dominated)
							
0.5	RDT	$341,288		99,875		$3	
	PT	$352,770	$11,482	99,772	103	$4	$111
	M	$396,926		99,864		$4	(Dominated)
							
0.6	PT	$340,114		99,808		$3	
	RDT	$345,110		99,888		$3	(Dominated)
	M	$399,605		99,877		$4	(Dominated)

#### Proportion of non-malaria febrile illness cases that werebacterial

Table 6 shows that RDT is more cost saving than the other diagnostic strategies even when the sensitivity is measured at different levels between 5% to 15%. Cost values for RDT increases with reduction in the proportion of NMFI and microscopy is dominated at all levels considered.

**Table 6:** Sensitivity Report by Proportion of NMFI cases that were bacterial

P3	Strategy	Cost	Incr Cost	Eff	Incr Eff	C/E	Incr C/E (ICER)
0.05	RDT	$339,091		99,913		$3	
	PT	$364,461	$25,370	99,845	68	$4	$374
	M	$395,788		99,905		$4	(Dominated)
							
0.075	RDT	$338,076		99,881		$3	
	PT	$365,064	$26,988	99,776	105	$4	$258
	M	$394,825		99,871		$4	(Dominated)
							
0.1	RDT	$337,060		99,849		$3	
	PT	$365,667	$28,607	99,708	142	$4	$202
	M	$393,861		99,838		$4	(Dominated)
							
0.125	RDT	$336,045		99,818		$3	
	PT	$366,269	$30,225	99,639	178	$4	$169
	M	$392,898		99,805		$4	(Dominated)
							
0.15	RDT	$335,029		99,786		$3	
	PT	$366,872	$31,843	99,571	215	$4	$148
	M	$391,934		99,772		$4	(Dominated)

#### RDT and microscopy sensitivity

ICER was found to sensitive to changes in RDT sensitivity (Table 7), but robust to changes in microscopy sensitivity (Table 8). RDT is cost-saving at the base level of 90% RDT sensitivity with costs increasing with rise in RDT sensitivity. Reduction in sensitivity of microscopy also led to a reduction in costs of microscopic diagnosis strategy. However, microscopy is dominated at all deterministic values of RDT and microscopy considered.

**Table 7:** Report by RDT Sensitivity

P5	Strategy	Cost	Incr Cost	Eff	Incr Eff	C/E	Incr C/E (ICER)
0.84	RDT	$336,244		99,858		$3	
	PT	$365,426	$29,182	99,735	123	$4	$237
	M	$394,247		99,851		$4	(Dominated)
							
0.87	RDT	$336,957		99,861		$3	
	PT	$365,426	$28,469	99,735	125	$4	$227
	M	$394,247		99,851		$4	(Dominated)
							
0.90	RDT	$337,670		99,863		$3	
	PT	$365,426	$27,756	99,735	127	$4	$218
	M	$394,247		99,851		$4	(Dominated)
							
0.94	RDT	$338,383		99,865		$3	
	PT	$365,426	$27,042	99,735	129	$4	$209
	M	$394,247		99,851		$4	(Dominated)
							
0.98	RDT	$339,096		99,867		$3	
	PT	$365,426	$26,329	99,735	131	$4	$200
	M	$394,247		99,851		$4	(Dominated)

**Table 8:** Report by Microscopy Sensitivity

P7	Strategy	Cost	Incr Cost	Eff	Incr Eff	C/E	Incr C/E (ICER)
0.75	RDT	$337,466		99,862		$3	
	PT	$365,426	$27,959	99,735	127	$4	$221
	M	$392,820		99,847		$4	(Dominated)
							
0.82	RDT	$337,466		99,862		$3	
	PT	$365,426	$27,959	99,735	127	$4	$221
	M	$393,890		99,850		$4	(Dominated)
							
0.85	RDT	$337,466		99,862		$3	
	PT	$365,426	$27,959	99,735	127	$4	$221
	M	$394,960		99,854		$4	(Dominated)
							
0.90	RDT	$337,466		99,862		$3	
	PT	$365,426	$27,959	99,735	127	$4	$221
	M	$396,030		99,857		$4	(Dominated)
							
0.96	RDT	$337,466		99,862		$3	
	PT	$365,426	$27,959	99,735	127	$4	$221
	M	$397,100		99,860		$4	(Dominated)

#### Adherence to ACT

As shown in Table 9, better adherence is associated with cost reduction for all diagnostic approaches with a corresponding decrease in the ICER. However, microscopy is also dominated at all the deterministic values used.

**Table 9:** Report by Adherence to ACT Sensitivity

P11	Strategy	Cost	Incr Cost	Eff	Incr Eff	C/E	Incr C/E (ICER)
0.4	RDT	$359,976		99,836		$4	
	PT	$390,437	$30,460	99,706	130	$4	$235
	M	$414,756		99,827		$4	(Dominated)
							
0.5275	RDT	$352,801		99,844		$4	
	PT	$382,464	$29,663	99,715	129	$4	$230
	M	$408,218		99,835		$4	(Dominated)
							
0.65	RDT	$345,626		99,852		$3	
	PT	$374,492	$28,866	99,725	128	$4	$226
	M	$401,681		99,843		$4	(Dominated)
							
0.80	RDT	$338,451		99,861		$3	
	PT	$366,520	$28,069	99,734	127	$4	$221
	M	$395,144		99,850		$4	(Dominated)
							
0.90	RDT	$331,276		99,869		$3	
	PT	$358,548	$27,272	99,743	126	$4	$216
	M	$388,607		99,858		$4	(Dominated)

### Costs of the individual diagnostic strategies

ICER was sensitive to changes in cost of RDT. With doubling of cost of RDT from $0.76 to $1.14, use of RDT option becomes less cost-effective than presumptive treatment at a malaria prevalence level of 40% (Table 10). Considering various prevalence levels, RDT was found to be less cost-effective at all prevalence levels above 30%. Despite doubling of costs of RDT, microscopy is found to be more costly at all malaria prevalence levels though not so when compared with presumptive treatment strategy.

**Table 10:** 50% rise in the cost of RDT

**0.2**	**RDT**	**$373,159**		**99,865**		**$4**	
	M	$388,888		99,826		$4	(Ext Dom)
	PT	$390,737	$17,578	99,662	202	$4	$87
							
0.3	RDT	$376,314		99,874		$4	
	PT	$378,081	$1,768	99,699	175	$4	$10
	M	$391,568		99,839		$4	(Dominated)
							
0.4	PT	$365,426		99,735		$4	
	RDT	$379,468		99,883		$4	(Dominated)
	M	$394,247		99,851		$4	(Dominated)
							
0.5	PT	$352,770		99,772		$4	
	RDT	$382,623		99,893		$4	(Dominated)
	M	$396,926		99,864		$4	(Dominated)
							
0.6	PT	$340,114		99,808		$3	
	RDT	$385,778		99,902		$4	(Dominated)
	M	$399,605		99,877		$4	(Dominated)

### Cost of ACT

ICER was very sensitive to a 50% rise in cost of ACT (Table 11). RDT remains more cost-effective than the other diagnostic options at a prevalence level of 40% though with an ICER value of 51,008 which is relatively very high. At malaria prevalence level of above 40%, RDT is more cost-effective than microscopic and presumptive diagnosis, and at levels below 40%, it still dominates both strategies. With a reduction in cost of ACT by half from $2.50 to $1.25, presumptive treatment will be more cost-effective than RDT and microscopy at malaria prevalence level of 40%. At lower prevalence rates of 20% and 30%, microscopy is dominated relative to presumptive treatment with ICER values of 313 and 411 respectively.

**Table 11:** Rise in cost of ACT by 50% point

P1	Strategy	Cost	Incr Cost	Eff	Incr Eff	C/E	Incr C/E (ICER)
0.2	RDT	$361,076		99,865		$4	
	M	$422,036		99,867		$4	(Dominated)
	PT	$512,996		99,977		$5	(Dominated)
							
0.3	RDT	$375,054		99,874		$4	
	M	$433,384		99,875		$4	(Dominated)
	PT	$500,683		99,974		$5	(Dominated)
							
0.4	RDT	$389,031		99,883		$4	
	M	$444,732	$55,701	99,882	1	$4	$51,008
	PT	$488,370		99,971		$5	(Dominated)
							
0.5	RDT	$403,009		99,893		$4	
	M	$456,080	$53,071	99,890	3	$5	$18,519
	PT	$476,057		99,968		$5	(Dominated)
							
0.6	RDT	$416,986		99,902		$4	
	PT	$463,744		99,965		$5	(Dominated)
	M	$467,428	$50,442	99,897	5	$5	$10,872

### Cost of antibiotics (amoxicillin)

As shown in Table 12, a reduction in the price of amoxicillin by 50% gave RDT more preferred values than the other diagnostic strategies for prevalence levels up to 60%. At the prevailing prevalence of 40%, RDT is less costly and microscopy is dominated, and at lower prevalence levels, microscopy is dominated by extended dominance. Unlike when there was decrease in the cost of amoxicillin, ICER was robust to a corresponding percentage increase in the cost of amoxicillin (Table 13). Microscopy was dominated at malaria prevalence levels of 40% or less, while presumptive treatment was found more cost-effective than other strategies at higher prevalence levels.

**Table 12:** Text report of reduced cost of Amoxicillin by 50% point

P1	Strategy	Cost	Incr Cost	Eff	Incr Eff	C/E	Incr C/E (ICER)
0.2	RDT	$301,037		99,953		$3	
	M	$360,833		99,938		$4	(Ext Dom)
	PT	$390,737	$89,700	99,662	291	$4	$309
							
0.3	RDT	$307,995		99,951		$3	
	M	$366,187		99,937		$4	(Ext Dom)
	PT	$378,081	$70,086	99,699	253	$4	$278
							
0.4	RDT	$314,952		99,950		$3	
	PT	$365,426	$50,473	99,735	214	$4	$235
	M	$371,540		99,935		$4	(Dominated)
							
0.5	RDT	$321,910		99,948		$3	
	PT	$352,770	$30,860	99,772	176	$4	$175
	M	$376,894		99,934		$4	(Dominated)
							
0.6	RDT	$328,867		99,946		$3	
	PT	$340,114	£11,247	99,808	138	$3	$81
	M	$382,247		99,933		$4	(Dominated)

**Table 13:** Text report of 50% rise in the cost of Amoxicillin

P1	Strategy	Cost	Incr Cost	Eff	Incr Eff	C/E	Incr C/E (ICER)
0.2	RDT	$356,389		99,953		$4	
	PT	$390,737	$34,348	99,662	291	$4	$118
	M	$415,001		99,938		$4	(Dominated)
							
0.3	RDT	$357,353		99,951		$4	
	PT	$378,081	$20,728	99,699	253	$4	$82
	M	$415,249		99,937		$4	(Dominated)
							
0.4	RDT	$358,316		99,950		$4	
	PT	$365,426	$7,109	99,735	214	$4	$33
	M	$415,496		99,935		$4	(Dominated)
							
0.5	PT	$352,770		99,772		$4	
	RDT	$359,280		99,948		$4	(Dominated)
	M	$415,744		99,934		$4	(Dominated)
							
0.6	PT	$340,114		99,808		$3	
	RDT	$360,243		99,946		$4	(Dominated)
	M	$415,991		99,933		$4	(Dominated)

## Discussion

The study demonstrates the cost-effectiveness of RDTs over microscopy and syndromic approach. The results are consistent with results from a previous study [28], which showed that RDTs are cost-effective relative to syndromic approach and microscopy. Also the results of the present study are similar with a study in lower level health facilities in Zambia, which was conducted within the actual malaria context using field-based data in a malarious population [30]. Surprisingly, microscopy malaria diagnosis was less effective than expected which goes to challenge the fact that microscopy is the gold standard for malaria diagnosis [32].

More than 50% of patients who were diagnosed as having malaria through the syndromic approach, and who may have received anti-malarials, turned out to be parasite-negative. Thus this study demonstrated that over-diagnosis and, therefore, over-prescription of anti-malarials, may be reduced through the use of RDTs among this population at health centres. Malaria over-diagnosis is still a major public health problem in Africa with studies suggesting between 50% and 99% of those prescribed anti-malarials being test-negative, depending on endemicity in the clinical setting [8,33-35]. The ability to rule out malaria can also lead to more opportune diagnosis and treatment of other causes of fever, such as acute respiratory infection, typhoid fever and meningitis and avoidance of the exposure of those without malaria to any side-effects of the drug and the restriction of anti-malarials to true test-positives.

Health workers might have altered their normal practice as a result of the study (Hawthorne effect), thus obeying test results and prescribing ACT only to those who are RDT positive. In real practice (absence of the research team), this may not be so, as studies have shown that anti-malarials are prescribed by health workers even if test results are negative [9,13]. Undoubtedly, this will affect the cost-effectiveness of RDTs. Therefore, it is important that policy makers make effort to encourage health workers to use the test results as a guide for treatment decisions.

The sensitivity analysis at various levels of prevalence stresses the relevance of RDT under a low prevalence level. However, as has been noted the better health outcome with RDT compared to presumptive treatment does not mean improved treatment of true malaria cases, since sensitivity of presumptive treatment is higher than that of RDT [28]. Instead, it demonstrates improvement on treatment of bacterial non-malarial febrile infection that could be treated inappropriately with ACT using presumptive treatment. Bacterial diseases are an important cause of avoidable deaths in children in Africa [36-38].

At higher probability levels RDT also showed to be more cost-effective compared to presumptive treatment and microscopy. The reason for this could be explained by the fact that higher NMFI implies low malaria prevalence, which in turn provides strong reason for diagnostic test before treatment. It also implies that if careful diagnosis is not carried out, there could be higher probability of giving malaria treatment to a patient with NMFI. This in turn has high cost implication, especially when the cost of ACT is considered. Again, at a low probability level of NMFI, presumptive treatment proves to be more cost-effective than RDT, while microscopy is dominated at all levels of NMFI cases. This implies that at low malaria prevalence, the probability that non-malarial febrile infection is bacterial should be important to decision makers.

Cost-effectiveness of RDT and other diagnostic tools also has much to do with adherence. The reason is because when someone adheres to treatment there will be less chances of going for second treatment option even though the cost implication in the case of NMFI may not be so much. NMFI also has cost-effectiveness impact on RDT treatment based on the fact that presumptive treatment may lead to antibiotics being given when a patient actually has malaria. Policy makers should, therefore, know that cost-effectiveness of RDT can be greatly reduced if there is poor ACT adherence level. In this study, patients adhered to ACT treatment up to 80% level. Improvement on this will further ensure a more effective result while relapse will negatively affect the gains of RDT over presumptive treatment and microscopy. In Nigeria, ACT is delivered free to children under five years in public health facilities and this is likely to ensure that adherence level to ACT is increased. However, it is worth noting that ACT adherence can decrease as result of patients' and caretakers forgetting to give the dose on certain days. They may also have incomplete dose if they perceive that they are cured after the initial few doses as have been noted in the past, when chloroquine was being used as the first-line anti-malarial drug [39]. Because of poverty, they may stop taking the drugs after one or two doses, to save tablets for later use by other members of the family.

A doubling of the cost of RDT made it less preferable to presumptive treatment. The implication of this is that policy makers should be more careful in choosing any policy that will cause an increase in the cost of RDT as consumers might become less willing to pay for the marginal increase in the cost. Again, a rise in the cost of RDT that is not checked by a corresponding decrease in the cost of ACT is likely to result in presumptive treatment being preferred over RDT and microscopy. In a low prevalence setting, such a situation could further worsen the challenges associated with inappropriate use of drugs including risk of resistance, further disease progression and consequent depletion of income. Thus, strategies must be in place to prevent increase in the cost of RDT if it is to remain cost-effective over other strategies especially at a declining prevalence level.

If the price of ACT rises at the current malaria prevalence level, treatment for malaria will be costly even for presumptive treatment with the cost of ACT significantly driving the cost-effectiveness of RDT. Such a finding has been noted elsewhere [28]. This calls for careful decision in fixing the price of ACT by any authority, as this could overshoot the societal ability and willingness to pay, and as well elude the cost containment of RDT. Considering the fact that Nigeria has a significant rural population, increasing the cost of ACT will make it further difficult for people to seek treatment at the appropriate time. It could also encourage presumptive treatment for two reasons: 1) RDT would no longer be affordable, and 2) microscopy with its high cost will not be affordable and available especially in the remote villages.

As cost of ACT is currently high, unnecessary spending on treatment that is not malaria can be avoided if RDT is used to ascertain the true health condition. This will enable health workers to treat malaria appropriately and at the same time avoid extra burden of second-line treatment cost.

On the other hand, a reduction in the cost of ACT which made presumptive treatment more cost-effective than the other options implies that consumers might end up preferring presumptive treatment strategy. Such a situation was prevalent in the era of chloroquine use, a very cheap anti-malarial drug. This means that with falling prices of ACT, efforts must be made to keep consumers informed of all the benefits of appropriate diagnosis of malaria, including the fact that ACT could face the same fate as chloroquine, if used inappropriately.

An incremental effect value that fluctuated drastically at the 40% prevalence level, which is close to the prevalence of 43.1% noted in this study, gives a clear picture of what was obtained in Nigeria before the change in malaria treatment regime. Before 2005, chloroquine was the first-line drug for malaria treatment. Given the low cost of chloroquine, people were given presumptive treatment on the assumption that an average Nigerian has malaria parasites. The regime change made the cost of treatment to rise, which simultaneously brought about the need to ensure that one has malaria parasite before treatment so as to avoid giving the costly ACT drug incorrectly. The analysis however shows the need for a reduced cost of ACT given that most people cannot afford the treatment cost. It also shows that lower price of ACT will go a long way to improving the treatment-seeking pattern of the people, especially the poor and vulnerable.

Although studies have investigated the cost-effectiveness of RDT in Africa [14,28-30], this study provides, the first estimates of the cost-effectiveness of RDT in Nigeria. A particular strength of this analysis is that costs are based on relevant information from the study. However, the results are also subject to a number of assumptions. The sensitivity analysis showed, that the overall cost-effectiveness of RDT is relatively robust to these assumptions. In terms of extrapolation to parts of Nigeria, Enugu east LGA is representative of areas of stable perennial malaria transmission in Nigeria.

The primary limitation of the study is that effectiveness was estimated from the results of the study where the study team delivered ACT treatment free of charge. In practice, availability of ACT may influence cost-effectiveness because patients may not be able to purchase ACT as a result of its high costs. However, this may not be necessarily so for under-fives, that are officially given free ACT in public facilities in Nigeria.

Secondly, all patients that were negative for malaria parasites were not given ACT. But in practice, health workers may not adhere strictly to this as evidence has shown that health workers are reluctant to refrain from treating for malaria after a negative test [9,16,40,41], a practice that is likely to reduce the cost-effectiveness of RDTs.

Thirdly, patients who were negative on microscopy or RDT received amoxicillin systematically. Realizing that not all these patients would require amoxicillin, they were subsequently refereed for further investigations and management and may have gotten proper treatment for their illnesses. This may have made RDTs more cost-effective and thus biased the cost-effectiveness estimates. However, the proportion of NMFI was too small as to have contributed to RDTs being more cost effective. Nevertheless, in actual practice, health workers may prescribe antibiotics to patients who are negative within the concept of integrated management of childhood illnesses and when this happens, it will even make RDTs more cost-effective.

Fourthly, clinical outcome rather than parasitological clearance was used to assess effectiveness of treatment. It is possible that parasitological clearance does not occur following treatment despite the non-recurrence of fever and this can influence the measure of effect since patients recorded as having recovered could still have malaria parasites. However, it is noteworthy that since fever is the major symptom that makes individuals seek health care, those who do not have fever within 21 days of treatment are unlikely to seek further care or incur additional expenditure on treatment, thus limiting the potential bias arising from use of clinical outcome for this analysis.

Finally, patients who reported prior anti-malarial drug intake were not included in the study as this could cause a possible bias considering the fact that having taken an anti-malarial drug, their test results may have read negative implying that they don't have malaria, whereas they may have had malaria prior to coming to the health centre. However, in this study, there were not a significant number of them.

## Conclusion

At the prevalence level of 43.1%, RDT was a cost effective strategy for diagnosis of malaria in Nigeria. Policy makers and healthcare providers can be confident that at the prevalence level of malaria in Nigeria, that it will be cost saving to use RDTs rather than a syndromic approach and microscopy. There is, therefore, increased opportunity for cost savings if RDTs are introduced in health facilities especially in rural communities where microscopic examination for malaria diagnosis is not readily available. RDTs decreased the number of false positive patients who would have received unnecessary medication and thus helped to prevent the erroneous treatment of fever caused by other infections, and may reduce the drug resistance of malaria. There are compelling reasons to justify the implementation of RDTs in Nigeria. Most of the health centres that serve the Nigerian poor do not have microscopes or trained technicians to examine blood films. Therefore, the reliance on these rapid tests, which are reliable, cheap, and simple enough to be used by non-laboratory staff, is likely to greatly contribute to an effective control of malariaThe wide application of RDT in Nigeria is also likely to avoid the treatment of patients with bacterial disease with the costly ACT and thus provide appropriate anti-bacterial treatment for those with bacterial infection.

## Competing interests

The authors declare that they have no competing interests.

## Authors' contributions

BSCU conceived and designed the study, BSCU, ENO, CAO and OEO collected the Data. BSCU, ENO, OEO and UG analysed the data. BSCU wrote up the manuscript with input from all the authors
